# A prospective study to evaluate the risk malignancy index and its diagnostic implication in patients with suspected ovarian mass

**DOI:** 10.1186/s13048-017-0351-2

**Published:** 2017-08-14

**Authors:** Santosh Kumar Dora, Atal Bihari Dandapat, Benudhar Pande, Jatindra Prasad Hota

**Affiliations:** Department of obstetrics and gynaecology, Veer Surendra Sai Institute of Medical Science And Research (VIMSAR), Burla, Sambalpur, Odisha India

**Keywords:** Risk malignancy index, Ovarian cancer, Ca-125, Adnexal mass

## Abstract

**Background:**

There is no universal screening method for discrimination between benign and malignant adnexal masses yet. Various authors have tried tumor markers, imaging studies, cytology but no one yet is a definite method for screening of cancer ovary, for which a combined diagnostic modality has come to practice in form of RMI. With this background we conducted our study “Evaluation of risk malignancy index and its diagnostic value in patients with adnexal masses”.

**Methods:**

The aim of the study was to determine the effectiveness of risk of malignancy index (RMI-3) in preoperative discrimination between benign and malignant masses and also to reveal the most suitable cut off value. We have conducted a prospective study between November 2014 to October 2016. We included the parameters like menopausal status, ultrasound features, and serum levels of tumor marker like CA-125 for calculating RMI 3. Then RMI was compared with the histopathological report which was taken as gold standard.

**Results:**

In the present study malignant tumors constitute 54.76% (69/126) & benign tumors 45.24% (57/126). Bilaterality in adnexal masses and multilocularity is higher in malignant tumors than benign tumor, but a P –value >0.005 failed to be proved significant in our study. Solid area is seen in 24.69% (20/81) of benign and 75.30% (61/81) of malignant tumor. Similarly ascites was found in 38.09% (48/126) of cases. Out of which 18.75% (9/48) cases were found to be benign and malignancy was confirmed in 81.25% (39/48) patients. There is statistically significant number of malignant ovarian cancer patients where ascites and solid area is seen in USG findings (*p* = 0.000). Risk of Malignancy Index compared with individual parameters of Ultrasound score, CA-125 or menopausal score and a cut-off point of 236 shows a very high sensitivity (72.5%), specificity (98.2%), positive predictive value (98.1%), negative predictive value (74.7%) and diagnostic accuracy (84.13%) for discriminating malignant and benign pelvic masses.

**Conclusion:**

Simplicity and applicability of the method in the primary evaluation of patients with pelvic masses makes it a good option in daily clinical practice in non-specialized gynecologic departments and also in developing countries where access to a gynaecologist oncologist is limited.

## Background

The presence of an adnexal mass is a frequent reason for a woman to be referred to a gynecologist. An adnexal mass may be benign or malignant. It is the risk of malignancy that propels us for early, accurate and prompt diagnosis to lessen mortality and morbidity. In India, ovarian cancer has emerged as the fourth most common malignancy among females with incidence varying between 5.4 and 8 per 100,000populations in different parts of the country [1]. As the symptoms of the ovarian cancer are very vague like bloating, pelvic or abdominal pain, poor appetite, feeling full quickly, and urinary urgency it is also known as “silent killer”. Thus, silent occurrence and slow progression, added to the fact that few effective methods for early diagnosis and no universal screening method for diagnosis of malignant ovarian tumor exists, made its mortality rate highest among gynecologic malignancies [2]. The main challenge is to identify patients with high-risk adnexal masses preoperatively and this is compounded by the lack of definitive noninvasive diagnostic test. The discrimination between benign and malignant adnexal mass is central to decision regarding clinical management and surgical planning in such patients. The standardize method for preoperative identification of probable malignant masses would allow optimization of first line treatment for women with ovarian cancer. Early identification of ovarian carcinomas and referral to a gyneco-oncologist can facilitate accurate staging of the disease and optimal cytoreductive treatment, enhancing patientsurvival [3, 4]. Currently clinical examination, ultrasound assessment, assay of tumor markers are part of standard work up for adnexal mass but none of these indicators alone is very sensitive or specific for detecting malignancy in ovarian masses.

To reduce the diagnostic dilemma between benign and malignant ovarian masses, a formula-based scoring system known as risk of malignancy index (RMI) was introduced by Jacobs et al. [5]. in 1990, which was term as RMI 1. It is a product of ultrasound findings (U), the menopausal status (M), and serum CA-125 levels (RMI = U X M X CA-125). The original RMI (RMI-1) has been modified in 1996 by Tingulstadet al. [6]. Known as (RMI2) and again in 1999 known as (RMI3) [7]. The difference between the new indices lies in the different scoring of ultrasound characteristics and menopausal status. The objective of our study is to assess the sensitivity and specificity of RMI 3 prospectively so that women with ovarian mass can be referred to an appropriate specialist.

## Methods

### Type of study

It was a prospective diagnostic study. The study period was from November 2014 to October 2016. All patients with ovarian mass admitted to the gynecology department of, VIMSAR, Burla, India were included in the study. A total of 126 patients were selected by using purposive sampling technique.

### Sampling unit

Each patient having an adnexal mass admitted to department of obstetrics & gynecology, VIMSAR, Burla for treatment.

### Ethical statement

The study was approved by the VIREC ethical committee of the hospital. The ethical committee approval number is 2014/P-I-RP/14 M–O-OBG036/032. The aim of the study was explained appropriately and informed written consent was obtained from all the patients.

### Clinical samples

Women already diagnosed cases of ovarian malignancy receiving chemotherapy, masses arising from urinary tract and gastrointestinal tract and pregnancy with its complications like ectopic, molar and post abortive were excluded from the study.

Information abstracted were age, parity, menstrual status, and family history of cancer, personal history of previous malignancies, symptoms and duration of symptoms. Leading symptoms such as abdominal mass, swelling/discomfort, abdominal pain, gastrointestinal symptoms, urinary symptoms, generalized malaise & fatigue were scrutinized.

All patients underwent routine physical examination. Particular attention was paid to breast examination, lymphadenopathy, abdominal examination and pelvic examination.

Besides the routine investigations, CA-125 serum levels, abdominal ultrasounds findings, and menopausal status of all the cases were recorded preoperatively.

The modified RMI (RMI 3) for each woman was calculated using the product of the ultrasound score (U), the menopausal score (M), and the absolute value of serum CA-125 inserted in the following formula:

$$ \mathrm{RMI}=\mathrm{UxMxserumCA}-125 $$

Five ultrasound features suggestive of malignancy were sought to derive U including multilocularity (more than bilocular), presence of solid areas, bilaterality, presence of ascites, and extra ovarian tumors or evidence of metastases. U of 1 was given if none or one of these findings was detected and a score of 3 if two or more of these features were present. Postmenopausal status was defined as more than one year of amenorrhea, or age older than 50 years for women who had undergone hysterectomy; they scored M = 3. All other patients who did not meet these criteria were defined in a premenopausal status which scored M = 1. The absolute values of serum CA-125 (U/ml) was entered directly into the mentioned equation. The histopathological diagnosis was considered as the gold standard for defining the outcomes. Hence, the RMI was evaluated for sensitivity, specificity, positive predictive value (PPV), negative predictive values (NPV) and diagnostic accuracy, with reference to the actual presence of a malignant or benign pelvic tumor.

Laparotomy was done in all cases. The type of surgical procedure done were either unilateral salpingo-oophorectomy, unilateral salpingo-ophorectomy with biopsy of the contralateral ovary, total abdominal hysterectomy and unilateral salpingoophorectomy, total abdominal hysterectomy with bilateral salpingo-oophorectomy, with ometectomy, with bilateral pelvic lymph node dissection and debulking surgery. Surgical staging was carried out in suspected malignant ovarian tumors. The pelvic and para-aortic lymph nodes were evaluated and all enlarged lymph node resected. Infracolic omentectomy was performed. The other operative findings that were recorded are gross appearance and cut surface, ascites, site of extra ovarian involvement and tumor size. The specimen was sent for histopathological study in the department of pathology VIMSAR Research, Burla. Tumors were classified according to World Health Organization definitions and malignant tumors were staged according to the criteria of the international Federation of Gynecology and Obstetrics (2014).

### Statistical analysis

All statistical analysis were done using SPSS version 24 (IBM) and Microsoft Excel 2016 for windows. A univariate statistical analysis was performed for all sonographic parameters and patient age. The Kologoromov-Smirnov test was used to evaluate the normal distribution of continuous data. According to their distribution, they were compared with the use of student’s t–test. The proportions of malignant and benign cases with different sonographic parameters were compared with chi-square, Fisher’s exact tests. To determine the best cut-off value to discriminate between benign and malignant adnexal masses, a receiver operating characteristics (ROC) curve was plotted and the odds ratio with 95% confidence interval was calculated. The best cut-off value was chosen according to the highest sensitivity with the lowest false-positive rate. A *P*-value <0.05 was considered to be significant.

## Results

During the period from November 2014 to October 2016 there were 126 patients presented with ovarian masses; those were diagnosed and operated at VSS institute of medical science and research, India. The Table 1 shows out of 126 cases studied, most common encountered were papillary serous cystadenocarcinoma 25.39% (32/126), followed by mucinous cystadenocarcinoma 11.9%(15/126), mucinous cystadenoma 15/126 (11.9%), and dermoid cyst 10.32% (13/126). In the present study malignant tumors constitute 54.76% (69/126) & benign tumors 45.24% (57/126). The surface epithelial tumors were the commonest constituting 79.4% (100/126) followed by the germ cell tumors 12.7% (16/126) and the sex-cord stromal tumors 2.4% (3/126). The detail characteristics of Age, USG score, menopausal status, serum CA 125 levels and RMI are summarized in Table 2. The average age of the patients with benign tumors was 37.12 ± 13.05 years, whereas for malignant tumors it was 47.30 ± 11.43 years. Below the age of 20 years total of 5.5% (7/126) ovarian tumors found. Out of which, 3.97% (5/126) were benign and 1.59% (2/126) were malignant in nature. Above the age of 60 years total of 10.32% (13/126) ovarian tumors found. Out of which 3.97% (5/126) were benign and 6.35% (8/126) were malignant in nature. There is a significant difference of mean age in years 47.30 ± 11.43 for malignant adnexal mass compared to 37.12 ± 13.05 years for benign adnexal mass with a *P*-value = o.ooo. Premenopausal patients predominate in our study with 61.1% (77/126) cases, while 38.89% (49/126) of the affected patients were in postmenopausal group. 62.34% (48/77) of the premenopausal patients had benign diseases, while 37.66% (29/77) had malignant diseases. Among the postmenopausal patients, 18.4% (9/49) had benign disease, while 81.6% (40/49) had malignant disease. In premenopausal age group most of the ovarian masses were benign compared to postmenopausal patients with a *P*-value of 0.000. The investigation revealed bilateral adnexal mass was found in 27.78% (35/126) of cases. Out of which 45.72% (16/35) cases were found to be benign and 54.28% (19/35) cases were found to be malignant confirmed by histopathological examinations. Bilaterality in adnexal masses is higher in malignant tumors than benign tumor, but a *P* –value 0.947 failed to be proved significant in our study.

**Table 1:** Distributions ovarian tumors according to histopathology

Sl. No.		Number	Percentage
Benign tumors			
01	Serous cystadenoma	12	9.5%
02	Serous cystadeno fibroma	1	0.8%
03	Papillary serous cystadenoma	7	5.5%
04	Mucinous cystadenoma	15	11.9%
05	Papillary mucinous cystadenoma	3	2.38%
06	Dermoid cysts	13	11.9%
07	Granulosa cell tumor	2	1.58%
08	Chocolate cyst	4	3.17%
	Total	57	45.24%
Malignant tumors			
01	Serous cystadeno carcinoma	10	7.9%
02	Papillary serous cystadeno carcinoma	32	25.4%
03	Mucinous cystadeno carcinoma	15	11.9%
04	papillary mucinous cystadeno carcinoma	5	3.96%
05	Dysgerminoma	2	1.58%
06	Yolk sac tumor	1	0.8%
07	Sertoli-leydig cell tumor	1	0.8%
08	Kruken berg tumor	3	2.38%
	Total	69	54.76%
	TOTAL (Benign + Mallignant)	126

**Table 2:** Distribution of cases according to Age, USG score, menopausal status, serum CA 125 levels and RMI

Parameter	Benign	Malignant	*p*-value
Age			
≤ 20	5 (3.97%)	2 (1.59%)	
20–39	26 (20.63%)	11 (8.7%)	
40–59	21 (16.7%)	48 (38.1%)	
≥ 60	5 (3.97%)	08 (6.35%)	
Menstrual status			
Premenopausal	48 (62.34%)	29 (37.66%)	0.000
Postmenopausal	9 (18.4%)	40 (81.6%)	0.000
USG characteristics			
Bilateral	16 (45.72%)	19 (54.28%)	0.947
Multilocular	29 (46.03%)	34 (53.97%)	0.859
Presence of solid areas	20 (24.69%)	61 (75.30%)	0.000
Presence of ascites	9 (18.75%)	39 (81.25%)	0.000
Presence of metastasis	1 (5.5%)	17 (94.4%)	0.000
USG SCORE			
1	35 (61.4%)	22 (38.6%)	0.001
3	22 (31.88%)	47 (61.12%)	
SerumCA-125(U/ml) (Mean ± Sd)	69.89 ± 44.10	502.09 ± 1525.09	0.001
RMI (Mean ± Sd)	109.06 ± 47.49	3534.57 ± 13,653.83	0.013
RMI ≥ 236	1 (1.75%)	56 (98.24%)	0.000
RMI <236	56 (74.66%)	19 (25.34)	

Multilocular lesions were found in 50% (63/126) cases. Out of which 46.03% (29/63) cases were found to be benign and 53.97% (34/63) cases were found to be malignant post-surgery with no statistical significance found in our case with a P –value 0.858. Presence of solid components was found in 64.28% (81/126) cases. Out of which 24.69% (20/81) cases were found to be benign and 75.30% (61/81) cases were malignant. Hence presence of solid components in adnexal masses is higher in malignant tumors than benign tumor as evidenced by a *P* –value 0.000 which is highly significant. Presence of ascites was found in 38.09% (48/126) of cases. Out of which 18.75% (9/48) cases were found to be benign and malignancy was confirmed in 81.25% (39/48) patients. Presence of ascites in adnexal masses is higher in malignant tumors than benign tumor (*P* –value = 0.000). Evidence of metastasis on USG was found in 14.28% (18/126) of cases. Out of which 5.5% (1/18) cases were found to be benign and 94.4% (17/18) cases were malignant.

We assigned scores of 1(absence of specific findings or presence of one finding), or 3 (two or more findings) to the subjects, depending on the ultrasound findings. 45.24% (57/126) cases had an ultrasound score of 1, while 54.76% (69/126) patients were scored 3. USG score 1 with benign tumor were found to be higher than those of malignant tumor (*P* –value = 0.000). USG score 3 with malignant tumor were found to be higher than those of benign tumor (*P* –value = 0.000). Of the 57 patients with an ultrasound score 1, 61.4% (35/57) had benign diseases, while 38.6% (22/57) had malignant diseases. 54.76% (69/126) patients in our series had an ultrasound score of 3, and among them, 31.88% (22/69) had benign, 61.12% (47/69) had malignant tumor. The mean value of CA-125 is (502.09 ± 1525.09) U/ml for malignant adnexal mass compared to (69.89 ± 44.10)U/ml benign masses (*p* value 0.000).

**Fig. 1:**
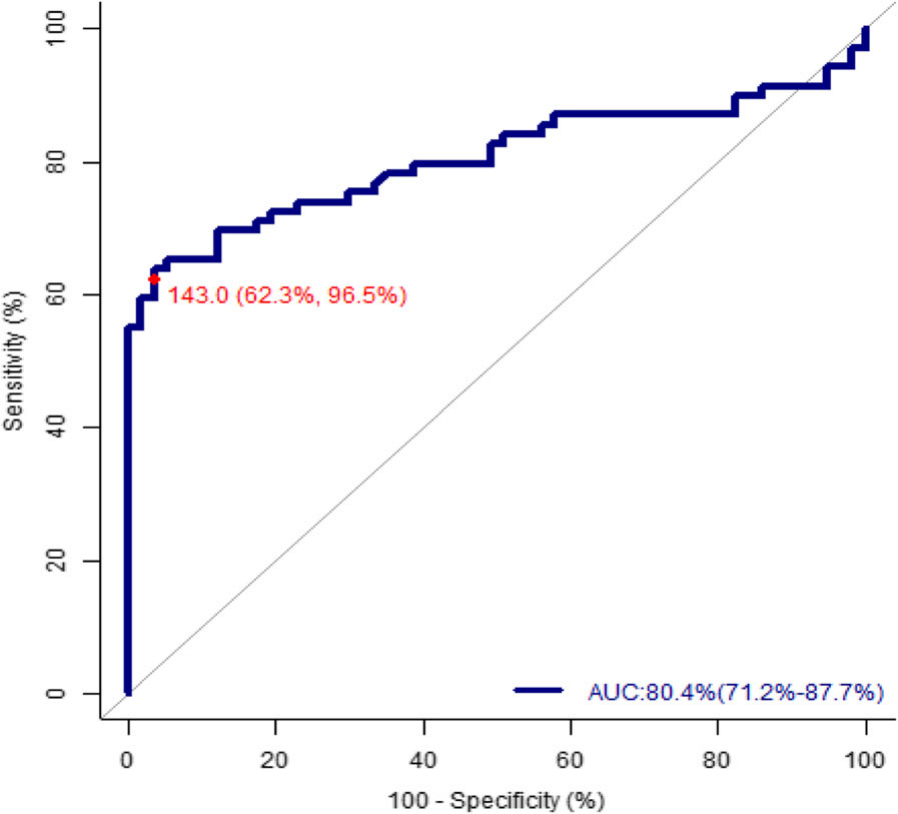
ROC curve of CA-125 in discriminating between benign and malignant adnexal masses

The performance of CA-125 and RMI (Table 3, Fig. 1) is shown in the receiver-operator characteristic curve (ROC). Best performance was obtained for serum CA-125 level of 143 U/ml (sensitivity 62.319%, specificity 96.491%, PPV 93.5%, NPV 67.5%,accuracy 77.78%,with highest area under the ROC curve i.e. 80.4%).The best performance obtained for RMI-3 was at the cut-off point 236 with a sensitivity of 72.5%, a specificity of 98.2%, a PPV of 98.1% an NPV of 74.7% and an accuracy of 84.13%.85.5% increase in the odds of diagnosing malignant adnexal masses with use of RMI when compared to not using RMI. Relative risk of diagnosing malignant adnexal masses 95.58% more with use of RMI when compared to not using RMI. 98% of malignant adnexal mass patients showing positive test result with RMI.75% of non-malignant adnexal mass patients showing negative test result with RMI. RMI ≥ 236 will increase the probability of diagnosing malignant adnexal masses from 54.8% to 98.15%.RMI < 236 will decrease the probability of diagnosing malignant adnexal masses from 54.8% to 22.24%. Diagnostic accuracy of RMI = 84.13%.Taking into account the best obtained cut-off point for RMI-3, 1 case was false positive (dermoid cyst) and 50 cases were true positive (RMI ≥236 malignant tumors) while 56 cases were true negative and 19 cases were false negative (RMI <236 malignant tumor);2 cases were dysgerminoma, 1 case was sertoli leydig cell tumor, 1 yolk sack tumor, 10 cases were serous cystadenocarcinoma, and 5 cases were mucinous cystadenocarcinoma.

**Table 3:** Evaluation of RMI, CA-125, USG score and menopausal status

	Sensitivity (%)	Specificity (%)	PPV (%)	NPV (%)	Accuracy (%)
RMI ≥ 236	72.5	98.2	98.1	74.7	84.13
CA-125 ≥ 143	62.3	96.5	93.5	67.5	77.77
USG Score 3	68.1	61.4	68.1	61.4	65.08
Menopause score 3	57.9	84.2	81.6	62.3	69.84

**Fig. 2:**
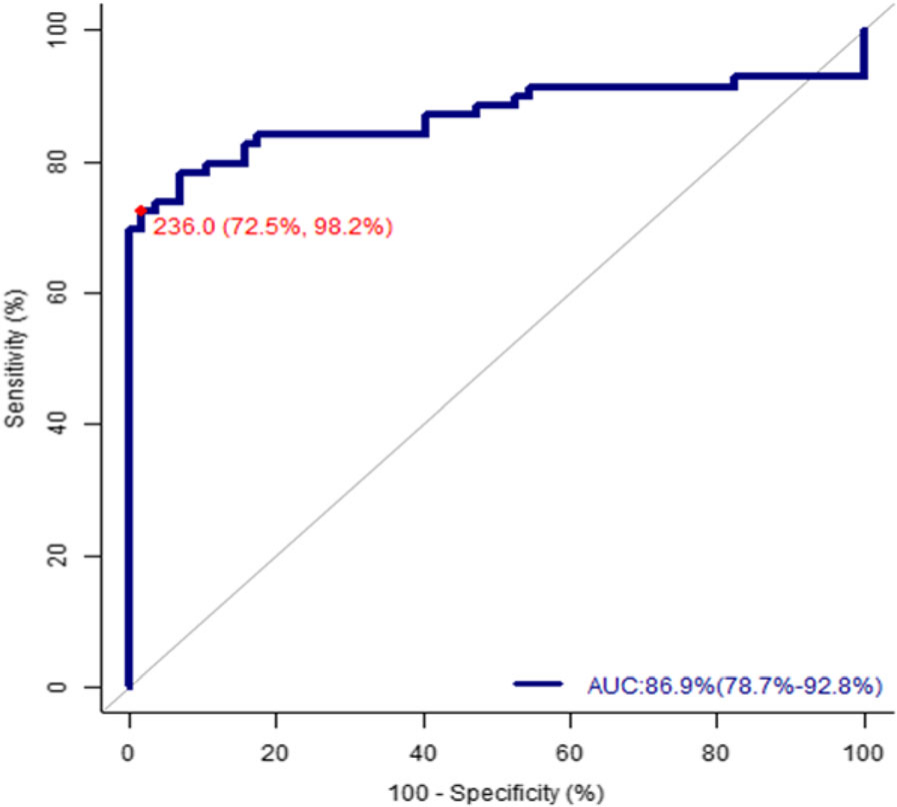
ROC curve showing the relationship between specificity and sensitivity for RMI-3 in differentiating between benign and malignant pelvic masses

We compared the diagnostic performance of RMI-3 score > 236, against CA-125 level > 143, ultrasound score of 3 and menopausal score of 3. Tables 4, 5 and Fig. 2 summaries the findings from this analysis. Among the criteria RMI score ≥ 236 has highest sensitivity, specificity, PPV, NPV, a diagnostic accuracy, when compared with individual parameters.

**Table 4:** Point estimates and 95% confidence interval of CA-125 at various cut off points

Cut off	True prevalence	Sensitivity (%)	Specificity (%)	PPV (%)	NPV (%)	+LR Ratio	-LR Ratio	Odds Ratio	Youden index	Accuracy(%)
≥35	54.8	87	19.3	56.6	55	1.078	0.676	1.594	0.063	56.35
≥50	54.8	84.1	42.1	63.7	68.6	1.452	0.379	3.835	0.262	65.08
≥100	54.8	72.5	78.9	80.6	70.3	3.442	0.349	9.868	0.514	76.19
≥143	54.8	62.319	96.491	93.5	67.5	11.841	0.398	29.769	0.571	77.78

**Table 5:** Point estimates and 95% confidence interval of RMI-3 at various cut off points

Cut off	True prevelance	Sensitivity (%)	Specificity (%)	PPV (%)	NPV (%)	+LR ratio	-LR Ratio	Odds ratio	Youden index	Accuracy(%)
150	54.8	79.7	84.2	85.9	77.4	5.048	0.24	20.952	0.639	77.78
153	54.8	79.7	84.2	85.9	77.4	5.048	0.24	20.952	0.639	77.78
200	54.8	73.9	96.5	96.2	75.3	21.06	0.27	77.917	0.704	84.13
236	54.8	72.5	98.2	98.1	74.7	43.78	0.23	185.5	0.751	84.13
238	54.8	72.5	98.2	98	74.7	41.36	0.28	147.36	0.707	84.13
265	54.8	69.3	98.2	98	72.7	39.65	0.31	128	0.678	82.54
300	54.3	69.3	98.3	98	73.1	40.34	0.31	130.28	0.678	83.33

## Discussion

About 10% of women undergo exploratory surgery for evaluation of ovarian masses during their lifetime [8]. Prompt identification of ovarian malignancies and referral to a gyneco-oncologist can enhance the patient survival rates [9]. but a single method which can accurately predict ovarian malignancy is still unavailable. In the pre-operative assessment of adnexal mass, the major diagnostic tools are still clinical impression and ultrasound examination. However, due to limitation of clinical impression and sonographic findings to predict ovarian malignancy, it is not surprising that gynecologists may detect an unexpected ovarian malignancy intra-operatively. Often an improper incision is made, the bowel is not adequately prepared or the surgeon is confronted with need to perform an unplanned cytoreductive surgery. A scoring system that predict ovarian malignancy can improve the chance of better preoperative counseling, better preoperative preparation and where appropriate referring the patients to a specialized center. Herein we report that the multiparametric RMI score can be a useful tool in prediction of malignant ovarian disease, in low-resource settings. Subsequent to introduction to RMI the same research group had reevaluated their diagnostic method in a new group of patients admitted for pelvic masses and confirmed the sensitivity and specificity of RMI and its priority compared to individual criteria [10]. The mean age of the patients with ovarian mass in our study was 42.69 years (range, 10 to 78 years). This is slightly higher than that reported in a similar study by Akdeniz*etal*. in 2009 [11]. In our study, 54.8% of the patients with an ovarian mass had malignant disease. Fifty eight percent of malignancies occurred in postmenopausal patients and 42% among the premenopausal patients. The data seem to agree with earlier reports of similar incidence rates and preponderance in postmenopausal patients [11, 12]. Rao (2014) has recently reported higher sensitivity, specificity, and positive and negative predictive values for a postmenopausal score of 3 [13]. In our study, this parameter had a higher specificity (84.2%) and positive predictive value (81.6%), but lower sensitivity (57.9%) and negative predictive values (62.3%) in assessing malignancy risk.

Ultrasonography is widely appreciated as the best imaging method for evaluation of ovarian pathology. Several groups have reported higher sensitivity, specificity, and positive predictive values for this method [9]. In our study, an ultrasound score of 3 had the sensitivity (68.1%), specificity 61.4%, positive predictive value 68.12% and negative predictive value (61.4%) among the parameters evaluated.

Several candidate biomarkers and their combinations have been employed in assessing the risk of ovarian malignancies, albeit with varying efficiency [14]. Serum CA-125 level is widely appreciated as a useful biomarker for estimating the risk of ovarian cancer, though other gynecological pathology can also increase its levels. Myers et al. [15]. have earlier reported sensitivity and specificity of less than 80%, for this marker, in the prediction of ovarian cancers. Simseket al. (2014) [16] has reported a sensitivity of 78.6% and specificity of 63.5% for a CA125 cut-off of 35 U/ml. Another report indicated a sensitivity of 88% and specificity of 97% for CA125 at a higher cut-off of 88 U/ml [12]. In our study, CA125 levels ≥35 U/ml had a sensitivity of 87%, specificity of only 19.3%, positive predictive value of 56.6%, and negative predictive value of 55% respectively. Best performance of CA-125 in our study was obtained at a cutoff of 143 with sensitivity 62.32%, specificity 96.49%, positive predictive value of 93.5% and negative predictive value of 67.5% and diagnostic accuracy of 77.77%. We suggest that a higher prevalence of inflammatory and nonspecific uterine and ovarian pathology, like pelvic inflammatory diseases and endometriosis might have contributed to elevated CA125 levels in the majority of our patients along with variable levels of CA125 regarding phases of menstrual cycles in premenopausal patients with adnexal masses and its more specificity for nonmucinousepithelial ovarian tumors account for its low diagnostic performance in the detection of malignant ovarian disease.

RMI is calculated from the serum CA125 antigen level, menopausal status, and ultrasonographic findings [5]. Several retrospective and prospective studies have reported it to be the best available tool for triage and referral of ovarian malignancies [10, 17]. Its utility as a diagnostic tool depends on the prevalence of malignancy in the study population [16]. We observed a high prevalence of malignancy (54.8%) among our study group, significantly higher than some of the earlier reports of 30–43% [5, 10, 17]. Jacobs et al. [5] (1990), studying 143 patients, reported a sensitivity of 85.4% and specificity of 96.9% for this method, with a cut-off of 200. Subsequently, several groups have reported its superior sensitivity and specificity in estimating the risk of ovarian malignancy, compared to other parameters. [7, 17–19]. The RMI cut-offs in many studies ranged from 25 to 250 (reviewed in Geominiet al. 2009) [18]. Most studies reported an increased diagnostic accuracy and performance with an RMI cut-off of 200 [5–7, 13, 19–23]. A recent study reported a sensitivity of 89.5%, specificity of 96.2%, positive predictive value of 77.3%, and negative predictive value of 98.4% [24] when a higher RMI cut-off of 238 was used for the screening. Yamamoto et al. (2009) [18] reported a sensitivity and specificity of 75% and 91%, respectively, using a cut-off of 450.The high sensitivity and specificity, PPV, NPV of the RMI at the optimal cut-off point of 236 in this study had a sensitivity of 72.5%, a specificity of 98.2% and a PPV of 98.1%, and an NPV of 74.7%. Bailey et al. [25] on 182 women with pelvic mass indicated an RMI > at a cut-off point of 200 with sensitivity of 88.5% for diagnosing the invasive lesions while Enakpeneet al. [26] on 302 women with pelvic mass indicated an RMI at a cut-off point of 250, a sensitivity of 88.2%, a specificity of 74.3%, a PPV of 71.3%, and an NPV of 90% for diagnosing the invasive lesions. In the current study, RMI at a cut-off point of 200 had a sensitivity of 73.9%, a specificity of 96.5% a PPV of 96.2%, and an NPV of 75.3%. According to Table 6, the results of previous studies described that many studies showed the best cut-off point for RMI was 200 [5–7, 10, 19–22, 27].

**Table 6:** Comparison of our results with previous studies

Study	No.	Sensitivity(%)	Specificity(%)	PPV(%)	NPV(%)
Jacob et al.1990	143	85.4	96.6		
Davies et al.1993	124	87.0	89.0		
Tingulstad et al.1996	173	71.0	96.0	89	88
Tingulstad et al.1999	365	71.0	92.0	69	92
Morgante et al.1999	124	58.0	95.0	78	87
Manjunath et al2001	152	73.0	91.0	93	67
Orres et al. 2002	154	73.0	86.0		
Ma et al. 2003	140	87.3	84.4	82	89
Andersen et al.2003	180	70.6	87.7	66	90
Ulusoy et al. 2006	236	71.7	80.5	67	84
Our study(RMI = 236)	126	72.5	98.2	98.1	74.7

A systematic review study by Geominiet al. [17] in 2009, 116 diagnostic studies for adnexal malignancy was reviewed. The reported result showed that RMI at cut-off point of 200 had a sensitivity of 78% and a specificity 87% for malignant mass diagnoses which was similar to our results.

According to the results of Ulusoys et al. in 2007, the RMI in a cut-off level of 153 showed a sensitivity of 76.4%, a specificity of 77.9%, a PPV of 65.9%, and an NPV of 85.5% for prediction of malignancy [19]. In the present study, RMI, at a cut-off level of 150 had a sensitivity of 79.7%, a specificity of 84.2%, a PPV of 85.9%, and an NPV of 77.4% for detection of malignancy. The best performance in the present study was seen with an RMI cut-off of 236, and the high sensitivity (72.5%) and high specificity (98.2%) observed were comparable to the majority of earlier reports that employed a similar cut-off.

Our results for RMI were in agreement with the results from other studies in which RMI was suggested to be better than other single parameters, with the highest area under the curve. In our study, RMI of ≥236 yielded high sensitivity, specificity, PPV, NPV, and accuracy of 72.5%, 98.2%,98.1%,74.7 and 84.13 respectively, which were similar compared with other studies.

At lower cut off values the sensitivity increases at the expense of specificity, while at a higher cut off values the specificity increases at the expense of sensitivity and more benign cases will be referred as malignant. So the decision of the cut off value (action line) will balance the sensitivity and specificity on one side and the local resources and availability of the specialists on the other side. When there is limitation of referral for specialist care because of distance resources, the RMI can be increased with some degree of sacrifice in sensitivity to achieve a higher level of specificity. In any scoring system to exclude malignancies, the false negative rate should ideally be zero or close to zero [28]..The present study observed nineteen false negative patients. Two cases were dysgerminoma, one case was sertoli leydig cell tumor, one yolk sack tumor, ten cases were serous cystadenocarcinoma, and five cases were mucinous cystadenocarcinoma. Ultrasound score is subjective, it relies on the expertise of the examiner. Gadducciet al. [29] reported mucinous tumors expressed CA-125 less than non-mucinous types. Besides low ultrasound score, the specificity of CA125 more for non-mucinous epithelial ovarian tumors are likely to explain the false negative results in the study.

## Conclusion

There is no universal screening method for discrimination between benign and malignant adnexal masses yet. So many authors have tried for earliest diagnosis of malignant ovarian tumors by various parameters. These may be earliest clinical features, tumor markers, imaging studies, cytology but no one yet is a definite method for screening of cancer ovary, In conclusion, the present study demonstrated that in the absence of a definite biomarker, the multi parametric Risk of Malignancy Index (RMI 3) was a better estimate in diagnosing adnexal masses with high risk of malignancy and subsequently guiding the patients to gynecological oncology centers for suitable and effective surgical interventions compared with individual parameters of Ultrasound score, CA-125 or menopausal score and a cut-off point of 236 shows a very high sensitivity (72.5%), specificity (98.2%), positive predictive value (98.1%), negative predictive value (74.7%) and diagnostic accuracy (84.13%) for discriminating malignant and benign pelvic masses. Simplicity and applicability of the method in the primary evaluation of patients with pelvic masses makes it a good option in daily clinical practice in non-specialized gynecologic departments. Besides in a low resource setting where sophisticated radiological and biochemical test may not be available at all places where RMI can be used as a investigations for the triage of patients and referral to a higher center.

## Abbreviations

M: Menopausal score
NPV: Negative predictive values
PPV: Positive predictive value
RMI: Risk of malignancy index
ROC: Receiver operating characteristics
U: Ultrasound score
